# The Abuse of Hypnotism

**Published:** 1890-09

**Authors:** 


### THE ABUSE OF HYPNOTISM.

THE theory of hypnotism has been discussed by men of all countries. Most wonderful reports of the power of suggestion are published every now and then from the European clinics, notably from Kraft-Ebing, Forel, Bernheim, and Charcot. A careful reading of these reports cannot fail to impress one with the thought that, in many instances, the operator, in his enthusiasm, forgets the true spirit of the duties of the medical man — to do, first, no harm to the patient; and, in the second place, to do him good if he can.

Many of the experiments are conducted, primarily, with the idea of investigating the field of hypnotism, to note how many suggestions can be given to a patient, and see how long they will operate. They seek not only to bring about useful results, but startling effects.

Thus, Charcot reports a patient hypnotised, to whom he suggested that a blank card was his photograph, and allowed the suggestion to remain in the post hypnotic state; thus bringing about the interesting condition of a person unable to distinguish a blank card, making him the laughing stock of the other inmates of the ward and ultimately leading the patient to distrust his own senses. The experiments of Kraft-Ebing, who hypnotised a nun and suggested that blisters and burns would appear on her body, did not do this for the patient's good, but to her positive harm. The experiment was interesting, but not justifiable. Conscious of their power, and glorying in it, men abuse it, as may be seen from this extract from Prof. Donato's article in the August *Cosmopolitan:*

I attended Madame Michéle, who had a stroke of paralysis. Her companion expressed doubts as to the efficacy of my curative power. Finally, somewhat irritated, I said: "Mademoiselle Léonie, to prove my power, I will paralyse your legs by a gesture. You can walk no more." In effect, Léonie, seized with fright, begged of me to give her back the use of her limbs.

This was as much assault and battery as if he had struck her, and was a vastly greater shock to her system. Just such frights can be the exciting cause of chorea and hysteria.

Donato says, further, that by surprising his patients he obtains paralysis and aphony. This is not for the patient's good.

Hypnotism has its therapeutic place, and it should not be used outside of it. If a patient has a disease or a symptom that can best be removed by hypnotism, by all means let it be used, but all temptation to make a show should be resisted, else this procedure will assuredly fall into disrepute, which it ought not to do.

